# Frontiers of myopia research in the 21st century: A bibliometric analysis of the top 100 most influential articles in the field

**DOI:** 10.1097/MD.0000000000040139

**Published:** 2024-10-18

**Authors:** Qi Xun, Wenjing Mei, Xuan Zhang, Yazheng Pang, Juan Yu

**Affiliations:** a School of Acupuncture-Moxibustion and Tuina, Shandong University of Traditional Chinese Medicine, Jinan, China; b Beijing First Hospital of Integrated Chinese and Western Medicine, Beijing, China; c Department of Children’s Massage Center, Affiliated Hospital of Shandong University of Traditional Chinese Medicine, Jinan, China.

**Keywords:** bibliometric analysis, CiteSpace, myopia, Scimago Graphica, the 21st century, top 100 most cited papers, trends, VOSviewer

## Abstract

Myopia is the most common refractive error worldwide, contributing not only to visual impairment but also serving as a potential risk factor for various severe ocular diseases. Its impact on patients’ quality of life and health is significant and imposes substantial socioeconomic burdens. In this study, we analyzed the top 100 most cited articles related to myopia published in the Web of Science Core Collection database from January 2000 to February 2024. Using data visualization tools CiteSpace, VOSviewer, and Scimago Graphica, we identified the most influential research papers and academic journals in the field of myopia. The top 100 most influential articles were published in 25 renowned journals across 30 countries, with *Investigative Ophthalmology & Visual Science* being the most prolific. *Ophthalmology* had the highest total citation frequency, while *The Lancet* had the highest average citation frequency. The United States and the National University of Singapore were the top countries and institutions with the most published papers. The 3 major research directions are Ophthalmology, Medicine, General & Internal, and Genetics & Heredity. The top 5 co-occurring keywords were refractive error, risk factors, prevalence, eye growth, and form-deprivation myopia. Cluster analysis results highlighted focal points such as retinal detachment, high myopia, and contrast sensitivity, indicating potential future research trends. Prospective research directions include investigating the pathogenesis of myopia, updating diagnostic technologies, and identifying risk genes for myopia and its complications.

## 1. Introduction

Myopia is characterized by ocular overdevelopment, where the elongation of the ocular axis prevents the precise convergence of distant parallel light rays onto the retina, resulting in compromised distance vision for affected individuals.^[1,2]^ As the most prevalent refractive error globally, the epidemiological landscape of myopia is of growing concern.^[3]^ Within the next 3 decades, half of the global population is expected to experience myopia, with the proportion of high myopia patients reaching up to 10%.^[4,5]^ Fueled by rapid technological advancements and lifestyle shifts, the prevalence of myopia continues to rise annually.^[6]^ The resulting visual impairments and decreased quality of life pose significant burdens on individuals and society, presenting a substantial challenge to global public health.^[7]^

The etiology of myopia is complex and involves factors such as genetic predisposition, environmental influences, and their interplay.^[8]^ Of particular concern is the heightened susceptibility of individuals with high myopia to severe visual impairment and associated risks of serious ocular complications, including glaucoma, cataracts, macular degeneration, and even blindness.^[9]^ Preventing and controlling myopia in children and adolescents are paramount due to their susceptibility as a high-risk population during critical periods of growth and development. Current research on the pathogenesis of myopia focuses on ocular structure, optical systems, and regulatory mechanisms of growth. Mainstream hypotheses include scleral remodeling, scleral hypoxia, reduced choroidal blood flow, inflammatory responses, and dopamine mechanisms.^[10–14]^ However, there are still uncertainties surrounding the underlying mechanisms of myopia. Current therapeutic strategies for managing myopia predominantly rely on pharmaceuticals, eyeglasses, surgical interventions, and behavioral modifications.^[15]^ Although these methods may control myopia progression to some extent, completely preventing its advancement remains a formidable challenge.^[16]^ Therefore, continuous research and exploration are necessary for this complex and enigmatic disease.

Bibliometric analysis stands as an effective method to offer researchers deeper insights into the trends and dynamics of a given domain.^[17]^ Regrettably, comprehensive and straightforward analysis reports on the research trends and prominent topics within the myopia field in the 21st century are currently lacking. In addition to the impact factor of journals, the citation count of an article serves as a critical measure of its contribution to a specific field. Therefore, we meticulously selected the top 100 most cited articles and employed bibliometric analysis to thoroughly examine the research landscape in the 21st-century myopia domain. This endeavor aimed to unveil the primary research directions within this field and to provide robust guidance and recommendations for resource allocation in future research efforts. We believe that this study will expedite progress in myopia research and provide a comprehensive and valuable reference for researchers worldwide.

## 2. Materials and methods

### 2.1. Data sources and search strategies

On March 18, 2024, we conducted a literature search on the Web of Science Core Collection (WoSCC) database (https://www.webofscience.com/wos/woscc/basic-search). The search criteria were as follows: TI = (myopia) OR TI = (myopic) OR TI = (myopias) OR TI = (nearsightedness) OR TI = (nearsightednesses) OR TI = (shortsightedness) OR TI = (near-sighted) OR TI = (short-sighted) OR TI = (high myopia) OR TI = (pathological myopia). The publication date range was set from January 1, 2000, to February 29, 2024. Publication types were limited to original articles and review articles, and the language was restricted to English.

### 2.2. Data screening strategies

**Figure 1.**
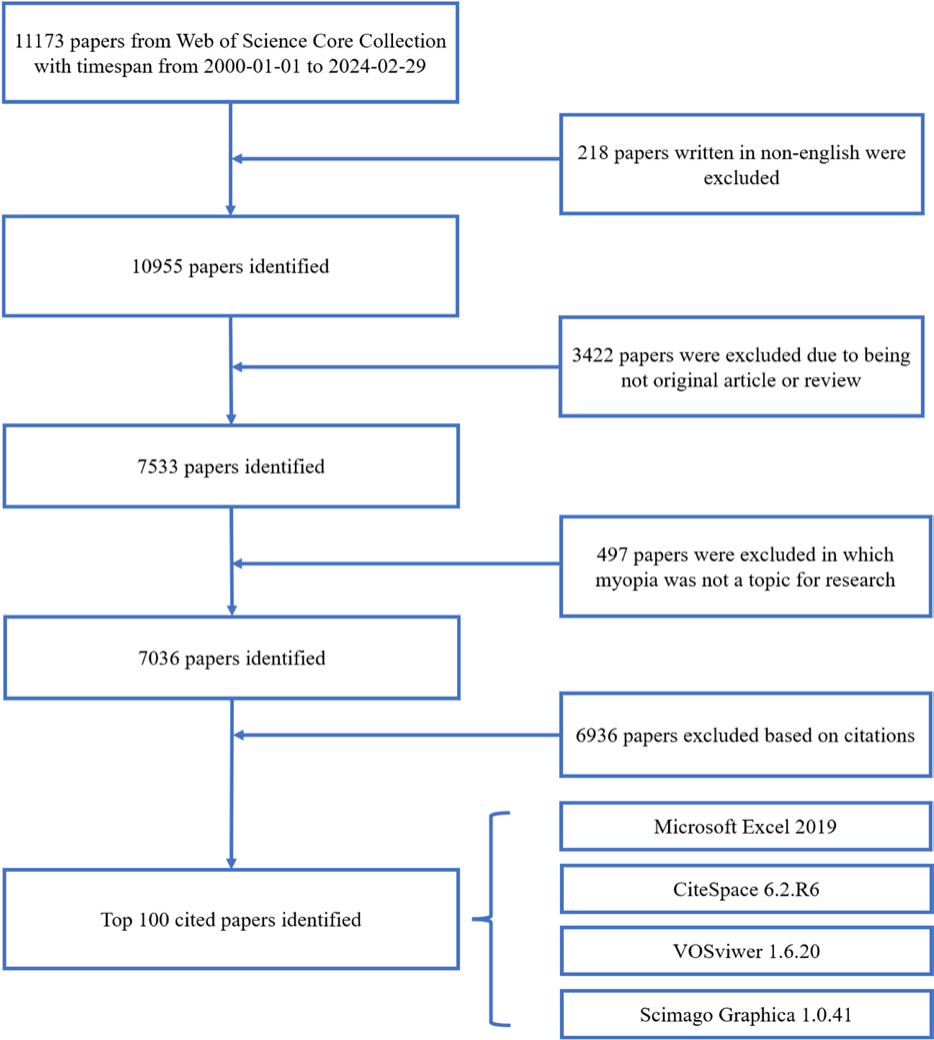
Literature screening process diagram.

Based on the frequency of citations, the literature obtained through the search strategy was sorted in descending order. The screening process was conducted independently by 2 researchers, who assessed the relevance of each document to the topic of myopia by reviewing the title, abstract, keywords, and full-text content, and initially excluded documents unrelated to myopia. When consensus could not be reached between the 2 researchers regarding the inclusion of an article, a third researcher was invited to participate in the discussion. The selection process is illustrated in Figure 1.

### 2.3. Data analysis strategies

We employed CiteSpace 6.2.R6, VOSviewer 1.6.20, and Scimago Graphica 1.0.41 to visualize article characteristics, enabling researchers to intuitively grasp research hotspots and knowledge dissemination pathways in the field of myopia. The study utilized VOSviewer to construct collaborative networks among countries, institutions, journals, and authors and to generate author density maps. Additionally, CiteSpace was used to track the flow of knowledge between disciplines at the journal level and to identify the most active and rapidly developing research topics within a specific time period.

## 3. Results

### 3.1. Citation characteristics of the included articles

Table 1 lists the top 100 cited papers, with a total of 35,741 citations (mean = 357.41). The most cited paper is “Global Prevalence of Myopia and High Myopia and Temporal Trends from 2000 through 2050” by Holden, BA (2255 citations), demonstrating the significance and impact of this paper in the field of myopia research. Following this are “Myopia” (1193 citations), “Outdoor activity reduces the prevalence of myopia in children” (805 citations), “Homeostasis of eye growth and the question of myopia” (728 citations), and “Myopia and associated pathological complications” (726 citations). There are 99 papers cited more than 200 times and 13 papers cited more than 500 times, further emphasizing the significant academic value of these works in the field of myopia research.

**Table 1:** The top 100 most cited papers in the field of myopia in the 21st century.

Rank	Title	First author	Journal	Total citation	Year	Average citation by year
1	Global prevalence of myopia and high myopia and temporal trends from 2000 through 2050	Holden, BA	OPHTHALMOLOGY	2255	2016	250.56
2	Ophthalmology 2 myopia	Morgan, IG	LANCET	1193	2012	91.77
3	Outdoor activity reduces the prevalence of myopia in children	Rose, KA	OPHTHALMOLOGY	805	2008	47.35
4	Homeostasis of eye growth and the question of myopia	Wallman, J	NEURON	728	2004	34.67
5	Myopia and associated pathological complications	Saw, SM	OPHTHALMIC AND PHYSIOLOGICAL OPTICS	726	2005	36.3
6	Prevalence of myopia in Taiwanese school children: 1983 to 2000	Lin, LLK	ANNALS ACADEMY OF MEDICINE SINGAPORE	616	2004	29.33
7	Enhanced depth imaging optical coherence tomography of the choroid in highly myopic eyes	Fujiwara, T	AMERICAN JOURNAL OF OPHTHALMOLOGY	611	2009	38.19
8	Small incision corneal refractive surgery using the small incision lenticule extraction (SMILE) procedure for the correction of myopia and myopic astigmatism: results of a 6 month prospective study	Sekundo, W	BRITISH JOURNAL OF OPHTHALMOLOGY	579	2011	41.36
9	Increased prevalence of myopia in the United States between 1971 to 1972 and 1999 to 2004	Vitale, S	ARCHIVES OF OPHTHALMOLOGY	578	2009	36.13
10	Effect of time spent outdoors at school on the development of myopia among children in China: A randomized clinical trial	He, MG	JAMA-JOURNAL OF THE AMERICAN MEDICAL ASSOCIATION	572	2015	57.2
11	The epidemics of myopia: Aetiology and prevention	Morgan, IG	PROGRESS IN RETINAL AND EYE RESEARCH	555	2018	79.29
12	Worldwide prevalence and risk factors for myopia	Pan, CW	OPHTHALMIC AND PHYSIOLOGICAL OPTICS	549	2012	42.23
13	Parental myopia, near work, school achievement, and children’s refractive error	Mutti, DO	INVESTIGATIVE OPHTHALMOLOGY & VISUAL SCIENCE	503	2002	21.87
14	How genetic is school myopia?	Morgan, I	PROGRESS IN RETINAL AND EYE RESEARCH	492	2005	24.6
15	The complex interactions of retinal, optical, and environmental factors in myopia etiology	Flitcroft, DI	PROGRESS IN RETINAL AND EYE RESEARCH	476	2012	36.62
16	Retardation of myopia in orthokeratology (ROMIO) study: A 2-year randomized clinical trial	Cho, P	INVESTIGATIVE OPHTHALMOLOGY & VISUAL SCIENCE	476	2012	36.62
17	International photographic classification and grading system for myopic maculopathy	Ohno-Matsui, K	AMERICAN JOURNAL OF OPHTHALMOLOGY	463	2015	46.3
18	Epidemiology and disease burden of pathologic myopia and myopic choroidal neovascularization: An evidence-based systematic review	Wong, TY	AMERICAN JOURNAL OF OPHTHALMOLOGY	449	2014	40.82
19	Atropine for the treatment of childhood myopia: Safety and efficacy of 0.5%, 0.1%, and 0.01% doses (atropine for the treatment of myopia 2)	Chia, A	OPHTHALMOLOGY	437	2012	33.62
20	High myopia and glaucoma susceptibility—The Beijing eye study	Xu, LA	OPHTHALMOLOGY	437	2007	24.28
21	Efficacy comparison of 16 interventions for myopia control in children: A network meta-analysis	Huang, JH	OPHTHALMOLOGY	427	2016	47.44
22	Role of the sclera in the development and pathological complications of myopia	McBrien, NA	PROGRESS IN RETINAL AND EYE RESEARCH	411	2003	18.68
23	A randomized clinical trial of progressive addition lenses versus single vision lenses on the progression of myopia in children	Gwiazda, J	INVESTIGATIVE OPHTHALMOLOGY & VISUAL SCIENCE	410	2003	18.64
24	The sclera and myopia	Rada, JAS	EXPERIMENTAL EYE RESEARCH	405	2006	21.32
25	Outdoor activity during class recess reduces myopia onset and progression in school children	Wu, PC	OPHTHALMOLOGY	396	2013	33
26	Myopia as a risk factor for open-angle glaucoma: A systematic review and meta-analysis	Marcus, MW	OPHTHALMOLOGY	393	2011	28.07
27	IMI—Defining and classifying myopia: A proposed set of standards for clinical and epidemiologic studies	Flitcroft, DI	INVESTIGATIVE OPHTHALMOLOGY & VISUAL SCIENCE	388	2019	64.67
28	Photodynamic therapy of subfoveal choroidal neovascularization in pathologic myopia with verteporfin: 1-year results of a randomized clinical trial: VIP report no. 1	Arnold, J	OPHTHALMOLOGY	387	2001	16.13
29	Atropine for the treatment of childhood myopia	Chua, WH	OPHTHALMOLOGY	382	2006	20.11
30	Ocular aberrations before and after myopic corneal refractive surgery: LASIK-induced changes measured with laser ray tracing	Moreno-Barriuso, E	INVESTIGATIVE OPHTHALMOLOGY & VISUAL SCIENCE	382	2001	15.92
31	Role of near work in myopia: Findings in a sample of Australian school children	Ip, JM	INVESTIGATIVE OPHTHALMOLOGY & VISUAL SCIENCE	367	2008	21.59
32	Refractive error, axial length, and relative peripheral refractive error before and after the onset of myopia	Mutti, DO	INVESTIGATIVE OPHTHALMOLOGY & VISUAL SCIENCE	358	2007	19.89
33	Nearwork in early-onset myopia	Saw, SM	INVESTIGATIVE OPHTHALMOLOGY & VISUAL SCIENCE	351	2002	15.26
34	Genome-wide meta-analyses of multiancestry cohorts identify multiple new susceptibility loci for refractive error and myopia	Verhoeven, VJM	NATURE GENETICS	347	2013	28.92
35	Long-term pattern of progression of myopic maculopathy: A natural history study	Hayashi, K	OPHTHALMOLOGY	345	2010	23
36	Prevalence, incidence, and progression of myopia of school children in Hong Kong	Fan, DSP	INVESTIGATIVE OPHTHALMOLOGY & VISUAL SCIENCE	331	2004	15.76
37	Five-year clinical trial on atropine for the treatment of myopia 2: Myopia control with atropine 0.01% eyedrops	Chia, A	OPHTHALMOLOGY	324	2016	36
38	Low-concentration atropine for myopia progression (LAMP) study: A randomized, double-blinded, placebo-controlled trial of 0.05%, 0.025%, and 0.01% atropine eye drops in myopia control	Yam, JC	OPHTHALMOLOGY	320	2019	53.33
39	Updates of pathologic myopia	Ohno-Matsui, K	PROGRESS IN RETINAL AND EYE RESEARCH	317	2016	35.22
40	Prevalence and progression of myopic retinopathy in an older population	Vongphanit, J	OPHTHALMOLOGY	313	2002	13.16
41	Outdoor activity and myopia in Singapore teenage children	Dirani, M	BRITISH JOURNAL OF OPHTHALMOLOGY	306	2009	19.13
42	United States Food and Drug Administration clinical trial of the Implantable Collamer Lens (ICL) for moderate to high myopia - 3-year follow-up	Sanders, DR	OPHTHALMOLOGY	305	2004	14.52
43	Laser in situ keratomileusis for myopia and astigmatism: Safety and efficacy—A report by the American Academy of Ophthalmology	Sugar, A	OPHTHALMOLOGY	303	2002	13.17
44	Verteporfin therapy of subfoveal choroidal neovascularization in pathologic myopia: 2-year results of a randomized clinical trial: VIP report no. 3	Blinder, KJ	OPHTHALMOLOGY	301	2003	13.68
45	Global variations and time trends in the prevalence of childhood myopia, a systematic review and quantitative meta-analysis: implications for etiology and early prevention	Rudnicka, AR	BRITISH JOURNAL OF OPHTHALMOLOGY	300	2016	33.33
46	Long-term effect of overnight orthokeratology on axial length elongation in childhood myopia: A 5-year follow-up study	Hiraoka, T	INVESTIGATIVE OPHTHALMOLOGY & VISUAL SCIENCE	296	2012	22.77
47	Prevalence of myopia and its association with body stature and educational level in 19-year-old male conscripts in Seoul, South Korea	Jung, SK	INVESTIGATIVE OPHTHALMOLOGY & VISUAL SCIENCE	293	2012	22.54
48	Time spent in outdoor activities in relation to myopia prevention and control: A meta-analysis and systematic review	Xiong, SY	ACTA OPHTHALMOLOGICA	288	2017	36
49	Corneal reshaping and myopia progression	Walline, JJ	BRITISH JOURNAL OF OPHTHALMOLOGY	288	2009	18
50	Incidence and progression of myopia in Singaporean school children	Saw, SM	INVESTIGATIVE OPHTHALMOLOGY & VISUAL SCIENCE	288	2005	14.4
51	The association between time spent outdoors and myopia in children and adolescents: A systematic review and meta-analysis	Sherwin, JC	OPHTHALMOLOGY	284	2012	21.85
52	Retinal and choroidal biometry in highly myopic eyes with spectral-domain optical coherence tomography	Ikuno, Y	INVESTIGATIVE OPHTHALMOLOGY & VISUAL SCIENCE	280	2009	17.5
53	Effect of dual-focus soft contact lens wear on axial myopia progression in children	Anstice, NS	OPHTHALMOLOGY	279	2011	19.93
54	Myopia prevention and outdoor light intensity in a school-based cluster randomized trial	Wu, PC	OPHTHALMOLOGY	278	2018	39.71
55	Time outdoors and physical activity as predictors of incident myopia in childhood: A prospective cohort study	Guggenheim, JA	INVESTIGATIVE OPHTHALMOLOGY & VISUAL SCIENCE	278	2012	21.38
56	Effect of myopia on the thickness of the retinal nerve fiber layer measured by cirrus HD optical coherence tomography	Kang, SH	INVESTIGATIVE OPHTHALMOLOGY & VISUAL SCIENCE	278	2010	18.53
57	Myopia, lifestyle, and schooling in students of Chinese ethnicity in Singapore and Sydney	Rose, KA	ARCHIVES OF OPHTHALMOLOGY	274	2008	16.12
58	Increasing prevalence of myopia in Europe and the impact of education	Williams, KM	OPHTHALMOLOGY	271	2015	27.1
59	The association between near work activities and myopia in children: A systematic review and meta-analysis	Huang, HM	PLOS ONE	270	2015	27
60	Influence of overnight orthokeratology on axial elongation in childhood myopia	Kakita, T	INVESTIGATIVE OPHTHALMOLOGY & VISUAL SCIENCE	266	2011	19
61	Retinal nerve fiber layer measurements in myopia: An optical coherence tomography study	Leung, CKS	INVESTIGATIVE OPHTHALMOLOGY & VISUAL SCIENCE	266	2006	14
62	Epidemiology of myopia	Foster, PJ	EYE	262	2014	23.82
63	Does education explain ethnic differences in myopia prevalence? A population-based study of young adult males in Singapore	Wu, HM	OPTOMETRY AND VISION SCIENCE	261	2001	10.88
64	Decrease in rate of myopia progression with a contact lens designed to reduce relative peripheral hyperopia: One-year results	Sankaridurg, P	INVESTIGATIVE OPHTHALMOLOGY & VISUAL SCIENCE	257	2011	18.36
65	The effect of ambient illuminance on the development of deprivation myopia in chicks	Ashby, R	INVESTIGATIVE OPHTHALMOLOGY & VISUAL SCIENCE	257	2009	16.06
66	First efficacy and safety study of femtosecond lenticule extraction for the correction of myopia—6-month results	Sekundo, W	JOURNAL OF CATARACT AND REFRACTIVE SURGERY	256	2008	15.06
67	Optical coherence tomography findings in myopic traction maculopathy	Panozzo, G	ARCHIVES OF OPHTHALMOLOGY	256	2004	12.19
68	An updated view on the role of dopamine in myopia	Feldkaemper, M	EXPERIMENTAL EYE RESEARCH	253	2013	21.08
69	Structural and ultrastructural changes to the sclera in a mammalian model of high myopia	McBrien, NA	INVESTIGATIVE OPHTHALMOLOGY & VISUAL SCIENCE	252	2001	10.5
70	Lamina cribrosa thickness and spatial relationships between intraocular space and cerebrospinal fluid space in highly myopic eyes	Jonas, JB	INVESTIGATIVE OPHTHALMOLOGY & VISUAL SCIENCE	248	2004	11.81
71	Interventions to slow progression of myopia in children	Walline, JJ	COCHRANE DATABASE OF SYSTEMATIC REVIEWS	238	2011	17
72	Prevalence and progression of myopic retinopathy in Chinese adults: The Beijing eye study	Liu, HH	OPHTHALMOLOGY	238	2010	15.87
73	A 3-year randomized clinical trial of MiSight lenses for myopia control	Chamberlain, P	OPTOMETRY AND VISION SCIENCE	237	2019	39.5
74	Time outdoors and the prevention of myopia	French, AN	EXPERIMENTAL EYE RESEARCH	236	2013	19.67
75	The complications of myopia: A review and meta-analysis	Haarman, AEG	INVESTIGATIVE OPHTHALMOLOGY & VISUAL SCIENCE	232	2020	46.4
76	Genome-wide analysis points to roles for extracellular matrix remodeling, the visual cycle, and neuronal development in myopia	Kiefer, AK	PLOS GENETICS	232	2013	19.33
77	Epidemiologic study of the prevalence and severity of myopia among school children in Taiwan in 2000	Lin, LLK	JOURNAL OF THE FORMOSAN MEDICAL ASSOCIATION	231	2001	9.63
78	Ocular optical aberrations after photorefractive keratectomy for myopia and myopic astigmatism	Seiler, T	ARCHIVES OF OPHTHALMOLOGY	231	2000	9.24
79	Myopia	Baird, PN	NATURE REVIEWS DISEASE PRIMERS	229	2020	45.8
80	Long-term follow-up of high myopic foveoschisis: Natural course and surgical outcome	Gaucher, D	AMERICAN JOURNAL OF OPHTHALMOLOGY	224	2007	12.44
81	Optical response to LASIK surgery for myopia from total and corneal aberration measurements	Marcos, S	INVESTIGATIVE OPHTHALMOLOGY & VISUAL SCIENCE	224	2001	9.33
82	Quantitative OCT angiography of the retinal microvasculature and the choriocapillaris in myopic eyes	Al-Sheikh, M	INVESTIGATIVE OPHTHALMOLOGY & VISUAL SCIENCE	222	2017	27.75
83	High prevalence of myopia and high myopia in 5060 Chinese university students in Shanghai	Sun, J	INVESTIGATIVE OPHTHALMOLOGY & VISUAL SCIENCE	222	2012	17.08
84	Progression of myopia in school-aged children after COVID-19 home confinement	Wang, JX	JAMA OPHTHALMOLOGY	221	2021	55.25
85	Myopia control with orthokeratology contact lenses in Spain: Refractive and biometric changes	Santodomingo-Rubido, J	INVESTIGATIVE OPHTHALMOLOGY & VISUAL SCIENCE	220	2012	16.92
86	Protective effects of high ambient lighting on the development of form-deprivation myopia in Rhesus monkeys	Smith, EL	INVESTIGATIVE OPHTHALMOLOGY & VISUAL SCIENCE	220	2012	16.92
87	RADIANCE: A randomized controlled study of ranibizumab in patients with choroidal neovascularization secondary to pathologic myopia	Wolf, S	OPHTHALMOLOGY	219	2014	19.91
88	Defocus Incorporated Soft Contact (DISC) lens slows myopia progression in Hong Kong Chinese schoolchildren: A 2-year randomized clinical trial	Lam, CSY	BRITISH JOURNAL OF OPHTHALMOLOGY	218	2014	19.82
89	Prevalence and characteristics of foveal retinal detachment without macular hole in high myopia	Baba, T	AMERICAN JOURNAL OF OPHTHALMOLOGY	216	2003	9.82
90	Risk factors for incident myopia in Australian school children: The Sydney adolescent vascular and eye study	French, AN	OPHTHALMOLOGY	215	2013	17.92
91	Patchy atrophy and lacquer cracks predispose to the development of choroidal neovascularisation in pathological myopia	Ohno-Matsui, K	BRITISH JOURNAL OF OPHTHALMOLOGY	215	2003	9.77
92	Nature and nurture: the complex genetics of myopia and refractive error	Wojciechowski, R	CLINICAL GENETICS	213	2011	15.21
93	Effects of foveal ablation on emmetropization and form-deprivation myopia	Smith, EL	INVESTIGATIVE OPHTHALMOLOGY & VISUAL SCIENCE	209	2007	11.61
94	The relationship between axial length and choroidal thickness in eyes with high myopia	Flores-Moreno, I	AMERICAN JOURNAL OF OPHTHALMOLOGY	208	2013	17.33
95	US food and drug administration clinical trial of the implantable contact lens for moderate to high myopia	Vukich, JA	OPHTHALMOLOGY	208	2003	9.45
96	IMI—Report on experimental models of emmetropization and myopia	Troilo, D	INVESTIGATIVE OPHTHALMOLOGY & VISUAL SCIENCE	206	2019	34.33
97	Macular retinoschisis in highly myopic eyes	Benhamou, N	AMERICAN JOURNAL OF OPHTHALMOLOGY	206	2002	8.96
98	Accommodation and related risk factors associated with myopia progression and their interaction with treatment in COMET children	Gwiazda, JE	INVESTIGATIVE OPHTHALMOLOGY & VISUAL SCIENCE	204	2004	9.71
99	Atropine for the treatment of childhood myopia: Changes after stopping atropine 0.01%, 0.1% and 0.5%	Chia, A	AMERICAN JOURNAL OF OPHTHALMOLOGY	201	2014	18.27
100	Scleral hypoxia is a target for myopia control	Wu, H	PROCEEDINGS OF THE NATIONAL ACADEMY OF SCIENCES OF THE UNITED STATES OF AMERICA	198	2018	28.29

COVID-19 = corona virus disease 2019, LASIK = laser-assisted in situ keratomileusis.

### 3.2. Year of publications and citations

**Figure 2.**
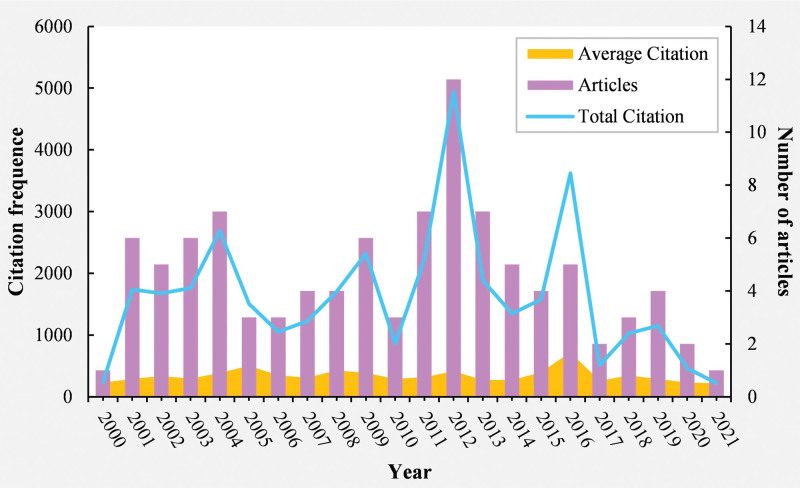
Year of publication and citation.

To illustrate the progression trajectory of myopia, we have constructed a chart (Fig. 2) based on the annual publication volume, average citation frequency, and total citation frequency of the included literature. This chart presents the top 100 most cited papers in the 21st century, published between 2000 and 2021. It is important to note that papers published from 2022 to 2024 are not included in this ranking. In 2016, the average citation frequency reached its peak (N = 724.6), reflecting the high quality and wide impact of papers published that year. Additionally, 2012 recorded the highest number of published papers (N = 12) and the highest total citation frequency (N = 4944). This suggests that 2012 represented a period of significant progress in myopia research, enhancing our understanding of myopia prevalence and risk factors, and fostering innovation in treatment and prevention methods.^[18–29]^

### 3.3. Distribution of countries/regions

**Figure 3.**
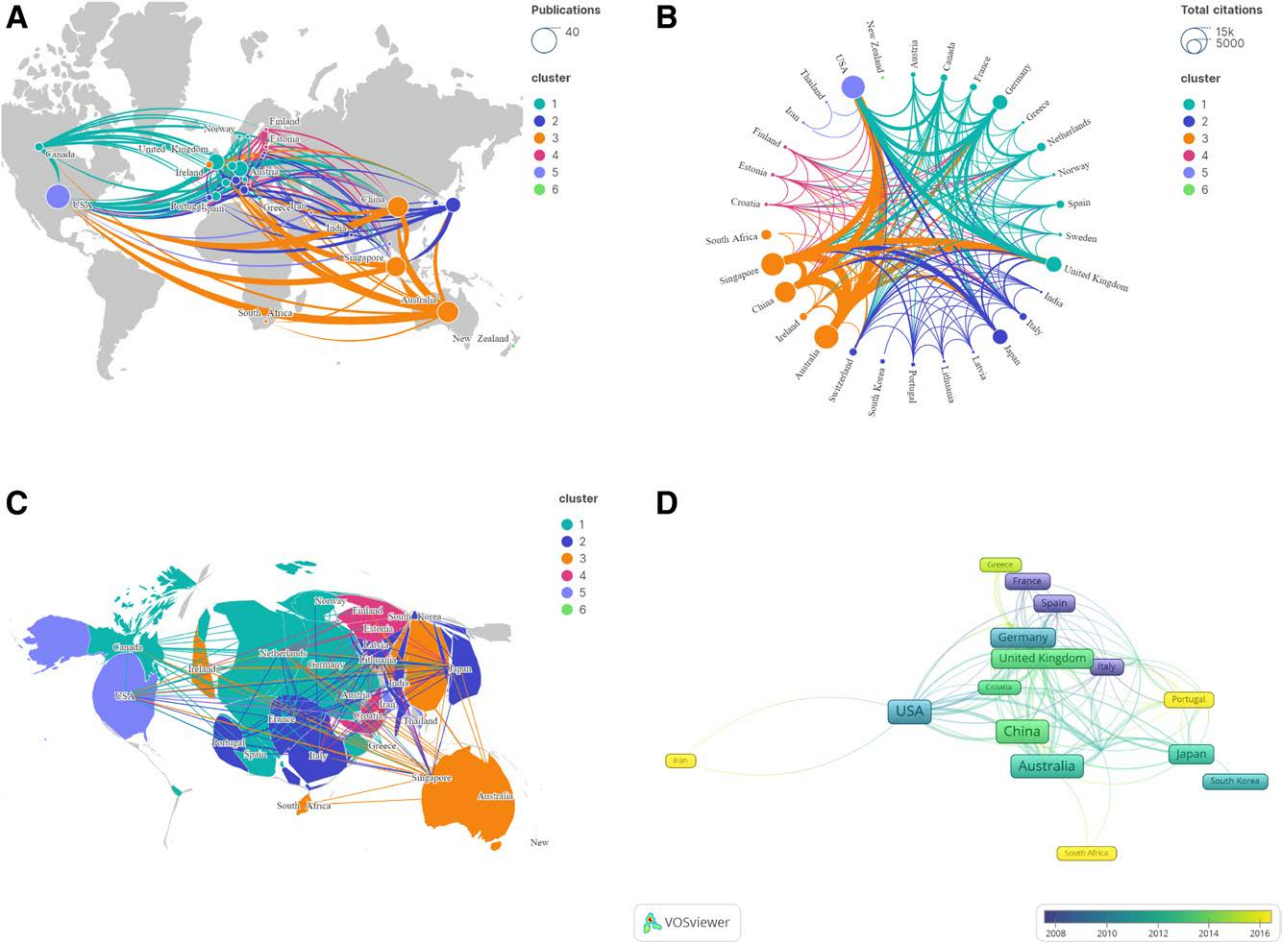
Collaboration network among countries/regions.

A total of 209 institutions in 30 countries/regions contributed to the research on the top 100 published papers. As shown in Figure 3A, the United States has the highest number of published papers (N = 34), followed by Australia (N = 26) and China (N = 25), ranking second and third, respectively. Among these 30 countries/regions, Australia achieves the highest number of citations (N = 12,013), followed by the United States (N = 11,164) and Singapore (N = 10,933) (Fig. 3B). These countries/regions demonstrated close collaboration, forming 6 major clusters (Fig. 3C). From the perspective of collaborative network coverage, the 3 largest country collaboration networks are centered around the United Kingdom, the United States, and Italy. Each network encompasses 21 countries, showcasing the extensive influence and deep collaboration of these nations in the field of myopia research. In recent years, Iran, Portugal, and South Africa have made contributions to research findings related to myopia (Fig. 3D). The inclusion of these emerging forces has undoubtedly injected new vitality into the field of myopia research.

### 3.4. Distribution of institutions

**Figure 4.**
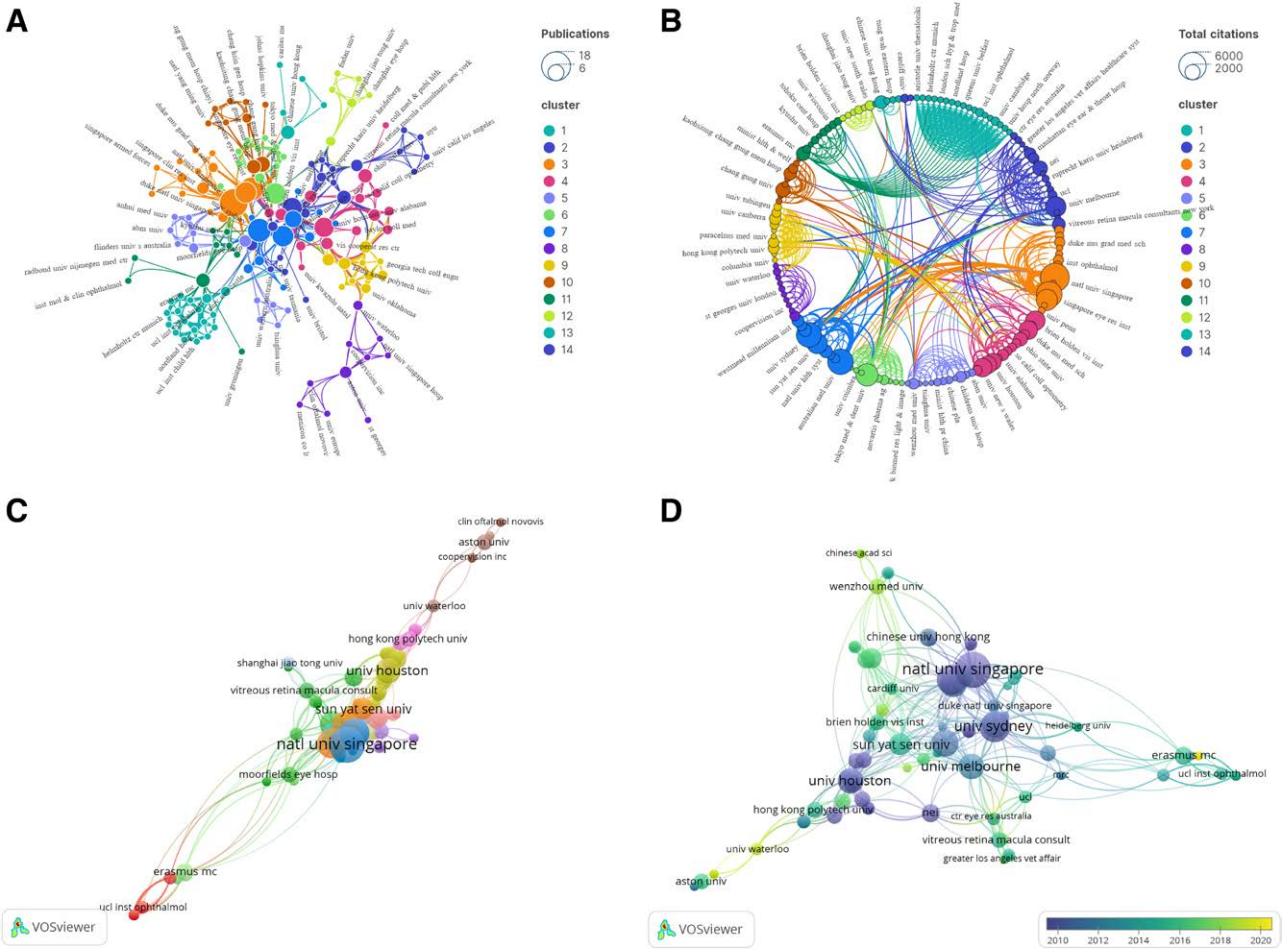
Institutional collaboration network of the top 100 most cited papers.

Regarding the number of publications, Natl Univ Singapore contributes the most papers (N = 16), followed by Singapore Eye Res Inst (N = 11) and Univ Sydney (N = 11) (Fig. 4A). In terms of citation frequency, Natl Univ Singapore stands out with the highest citation count (N = 5929), trailed by Singapore Eye Res Inst (N = 4753) and Australian Natl Univ (N = 4709) (Fig. 4B). The largest institutional collaboration network is led by Natl Univ Singapore, encompassing 51 institutions. Tokyo Med & Dent Univ and Univ Melbourne follow suit, covering 40 and 33 institutions respectively, highlighting the proactive role of these institutions in fostering collaboration in myopia research. Figure 4C shows that a total of 151 institutions were integrated into 14 main clusters. Natl Univ Singapore plays a crucial bridging role in the collaborative network, facilitating communication and cooperation among institutions. As shown in Figure 4D, in recent years, Chang Gung Univ, Kaohsiung Chang Gung Mem Hosp, and Wenzhou Med Univ have shown outstanding performance in terms of publications, emerging as new forces in this field.

### 3.5. Distribution of journals

**Figure 5.**
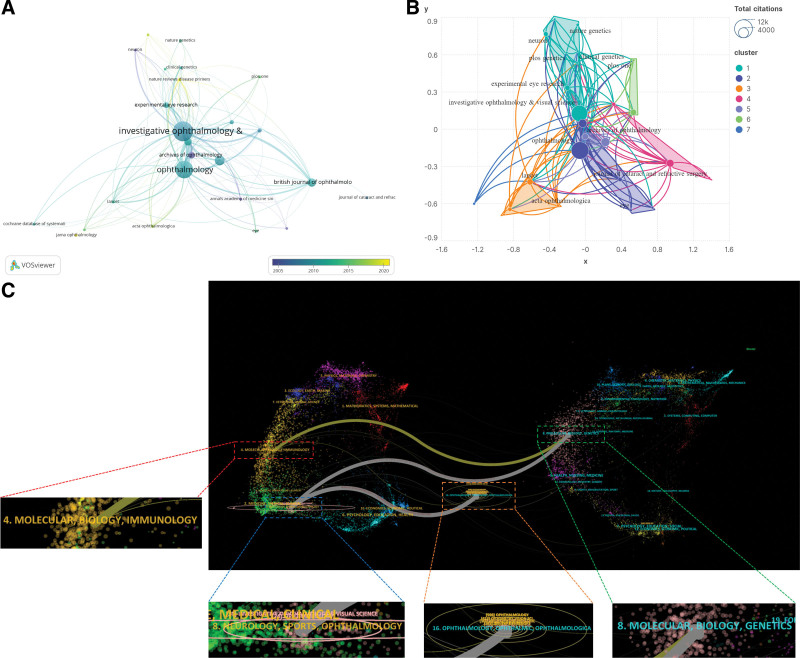
Visualization of the journals that contributed to the top most 100 cited papers.

The top 100 most cited papers were published in 25 different journals, with professional journals accounting for the majority (88%) of these publications. Among these, *Investigative Ophthalmology & Visual Science* has the highest publication volume (N = 30), followed by *Ophthalmology* (N = 24) (Fig. 5A). The journal with the highest total citation frequency *is Ophthalmology* (N = 10,122) (Fig. 5B). In contrast, *The Lancet* has the highest average citation frequency (1 paper, 1193 citations). The journal published a comprehensive review article titled “Myopia,” covering various aspects of myopia such as biological basis, definition, epidemiological characteristics, pathological mechanisms, risk factors, and various intervention measures. This review article provides valuable references and insights for scholars in the field of myopia research.^[18]^ To further investigate the dynamics of knowledge dissemination among journals in the myopia research field, we constructed a dual-overlay journal map (Fig. 5C). The left panel illustrates the primary clusters of journals prevalent in this domain, while the right panel elucidates the major clusters of cited journals. This map distinctly identifies 3 primary citation pathways, suggesting that articles published in OPHTHALMOLOGY and MOLECULAR/BIOLOGY/GENETICS journals are frequently cited by those published in MOLECULAR/BIOLOGY/IMMUNOLOGY and NEUROLOGY/SPORTS/OPHTHALMOLOGY journals. This finding provides valuable resources for conducting high-quality literature retrieval and exploring new research avenues.

### 3.6. Author and coauthor analysis

**Figure 6.**
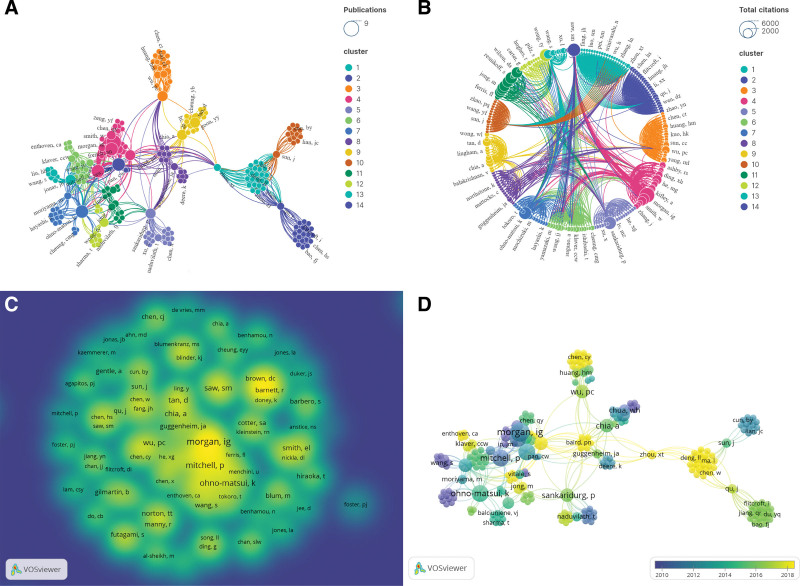
Network visualization of authors that contributed to the top most 100 cited papers.

A total of 544 authors contributed to the top 100 most cited papers. To avoid confusion caused by author name abbreviations, we standardized the format of authors’ names. For instance, “Rose, Kathryn A.” was normalized to “Rose, KA.” From Figure 6A, it can be seen that Morgan, IG had the highest number of publications (N = 8), followed by Saw, SM (N = 6), Rose, KA (N = 6), Mitchell, P (N = 6), and Ohno-Matsui, K (N = 6). The total connectivity strength of the author network centered around Ohno-Matsui, K was 52, and the largest author cluster in this domain comprised 49 authors. The most cited author was Morgan, IG (N = 4217), closely followed by Saw, SM (N = 3075), and Sankaridurg, P (N = 3029) (Fig. 6B). However, Fricke, TR, Holden, BA, Naduvilath, TJ, Naidoo, KS, and Wilson, DA had the highest average citations per paper (1 paper, 2255 citations). As shown in Figure 6C and D, Morgan, IG stands as a leading figure in the field of myopia research, while Enthoven, CA, Baird, PN, Matsui, KO, and Ding, G have emerged as promising scholars in recent years.

### 3.7. Research direction

According to the Web of Science categories, the literature included in this study was classified into various research topics (Table 2). The most mainstream research direction was “Ophthalmology” (N = 88). Within this category, the most extensively studied subjects were epidemiological surveys on myopia (N = 32) and myopia treatments (N = 24), which collectively accounted for more than half of the top 100 papers. This emphasizes the critical importance of these 2 major themes in myopia research.

**Table 2:** WOS categories in the top 100 cited papers on myopia.

WoS categories	Number
Ophthalmology	88
Medicine, General & Internal	6
Genetics & Heredity	3
Multidisciplinary Sciences	2
Surgery	1
Neurosciences	1

The scope of epidemiological investigations covered various countries and regions, including the United States, South Korea, Singapore, Australia, and regions in China such as Shanghai, Hong Kong, and Taiwan. This comprehensive coverage fully demonstrates the significance and universality of the myopia issue on a global scale. The risk factors that received the most attention in research included outdoor activities, near work, light intensity, educational pressure, and parental history. In research on myopia treatment, 4 studies focused on laser-assisted in situ keratomileusis and photorefractive keratectomy, one on small incision lenticule extraction, 2 on implantable collamer lenses, 5 on corneal reshaping procedures, and 5 on atropine eye drops. These diverse treatment methods provide myopic patients with more options, reflecting the activity and innovation within the field of myopia treatment. Additionally, “Medicine, General & Internal” (N = 6) was considered a significant research domain.

### 3.8. Keywords co-occurrence, clusters, and bursts

Co-occurrence analysis was performed using CiteSpace 6.2.R6 software, with a threshold set at K = 25. After merging similar keywords, a total of 322 keywords were identified from the top 100 most cited papers. These keywords accurately represent the research hotspots and core topics in the field of myopia. Table 3 summarizes the top 10 most frequently occurring keywords as follows: refractive error (N = 47), risk factors (N = 26), prevalence (N = 21), eye growth (N = 19), form-deprivation myopia (N = 18), children (N = 18), progression (N = 17), eye (N = 12), population (N = 12), and eye (N = 12).

**Table 3:** Top 10 co-occurring keywords in the most cited 100 papers on myopia.

Rank	Keywords	Occurrences
1	Refractive error	47
2	Risk factors	26
3	Prevalence	21
4	Eye growth	19
5	Form-deprivation myopia	18
6	Children	18
7	Progression	17
8	School children	15
9	Population	12
10	Eye	12

**Figure 7.**
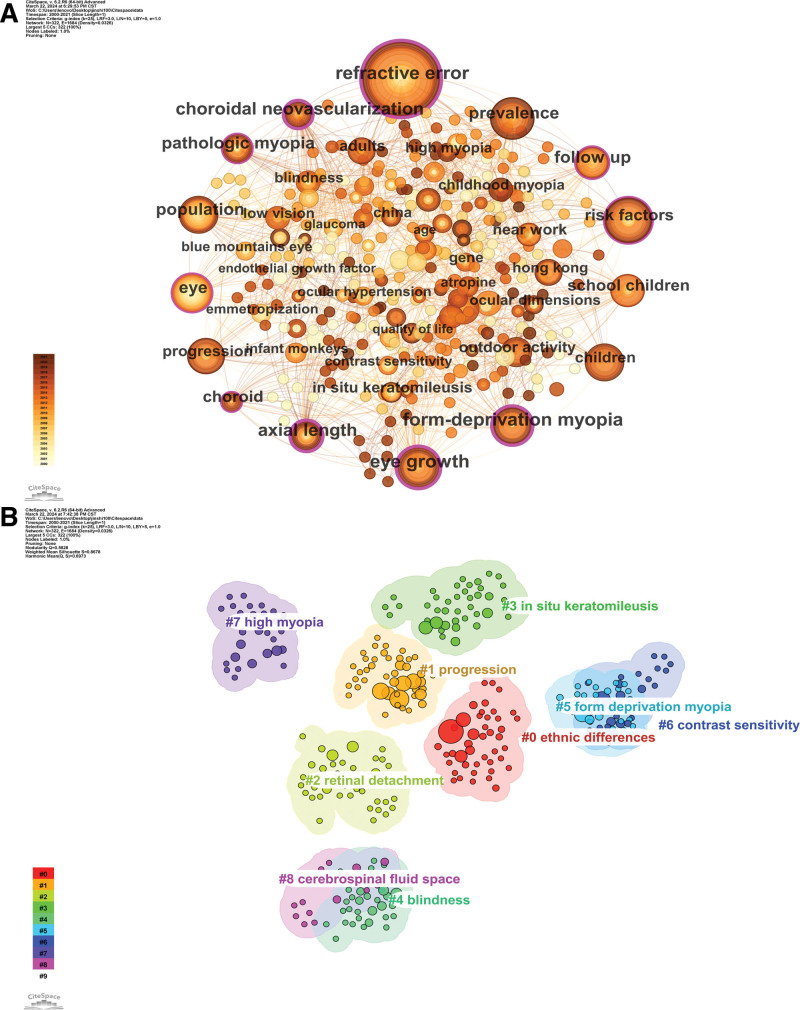
Network visualization and cluster analysis of co-occurring keywords in the top 100 most cited papers.

Myopia is considered to result from the combined influence of various factors. Most scholars believe that the etiology of myopia primarily involves 2 factors: environmental and genetic (N = 4). Among them, environmental factors include outdoor activity (N = 9), near work (N = 7), ambient illuminance (N = 3), and education (N = 3). The most frequently concerned epidemiological contents include prevalence (N = 21), population (N = 12), ethnic differences (N = 5), and age (N = 4). China (N = 6), Hong Kong, China (N = 8), and Australia (N = 2) are the regions most frequently involved in population surveys on myopia incidence. The disease groups receiving the most attention include children (N = 18), adults (N = 7), and older population (N = 3), with a particular focus on school children (N = 15). Currently, the most commonly used medications for myopia treatment are atropine (N = 6) and pirenzepine ophthalmic gel (N = 6), while the most frequently employed surgical methods are in situ keratomileusis (N = 6) and photorefractive keratectomy (N = 3). Progression of myopia may lead to serious complications, including choroidal neovascularization (N = 8), glaucoma (N = 3), macular degeneration (N = 3), cataract (N = 3), rhegmatogenous retinal detachment (N = 2), and retinal detachment (N = 2); in severe cases, it may result in blindness (N = 5). The most commonly used clinical observation indicators include axial length (N = 9), contrast sensitivity (N = 3), ocular refraction (N = 3), and corneal curvature (N = 2). Outcome endpoints of particular concern include eye growth (N = 19), progression (N = 17), follow-up (N = 10), and quality of life (N = 4). The specific types of myopia most commonly reported are form-deprivation myopia (N = 18), pathologic myopia (N = 9), and high myopia (N = 4) (Fig. 7A).

The research hotspots and main research directions in the field of myopia can be delineated through the analysis of high-frequency co-occurring keyword clusters (Fig. 7B). The largest red cluster primarily focuses on the etiological factors of myopia, encompassing racial differences, ocular biological characteristics, family history, and prolonged near work. The orange cluster, by contrast, is closely associated with the progression of myopia. The yellow cluster primarily focuses on the serious consequences of myopic changes, such as retinal detachment, optic nerve changes, and spontaneous decomposition. Meanwhile, the green cluster concentrates on the treatment modalities for myopia, including procedures such as in situ keratomileusis, intraocular lens implantation, and correction of high myopia. These keywords are further subdivided into 10 clusters as follows: Cluster 1 (ethnic differences), Cluster 2 (progression), Cluster 3 (retinal detachment), Cluster 4 (in situ keratomileusis), Cluster 5 (blindness), Cluster 6 (form deprivation myopia), Cluster 7 (contrast sensitivity), Cluster 8 (high myopia), Cluster 9 (cerebrospinal fluid space), and Cluster 10 (limits).

**Figure 8.**
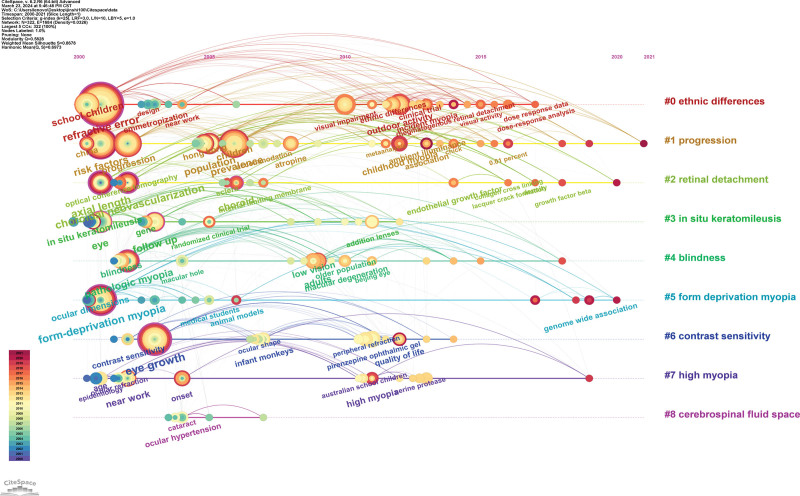
Visualization of clustering analysis of co-occurring keywords in the top 100 most cited papers over time.

**Figure 9.**
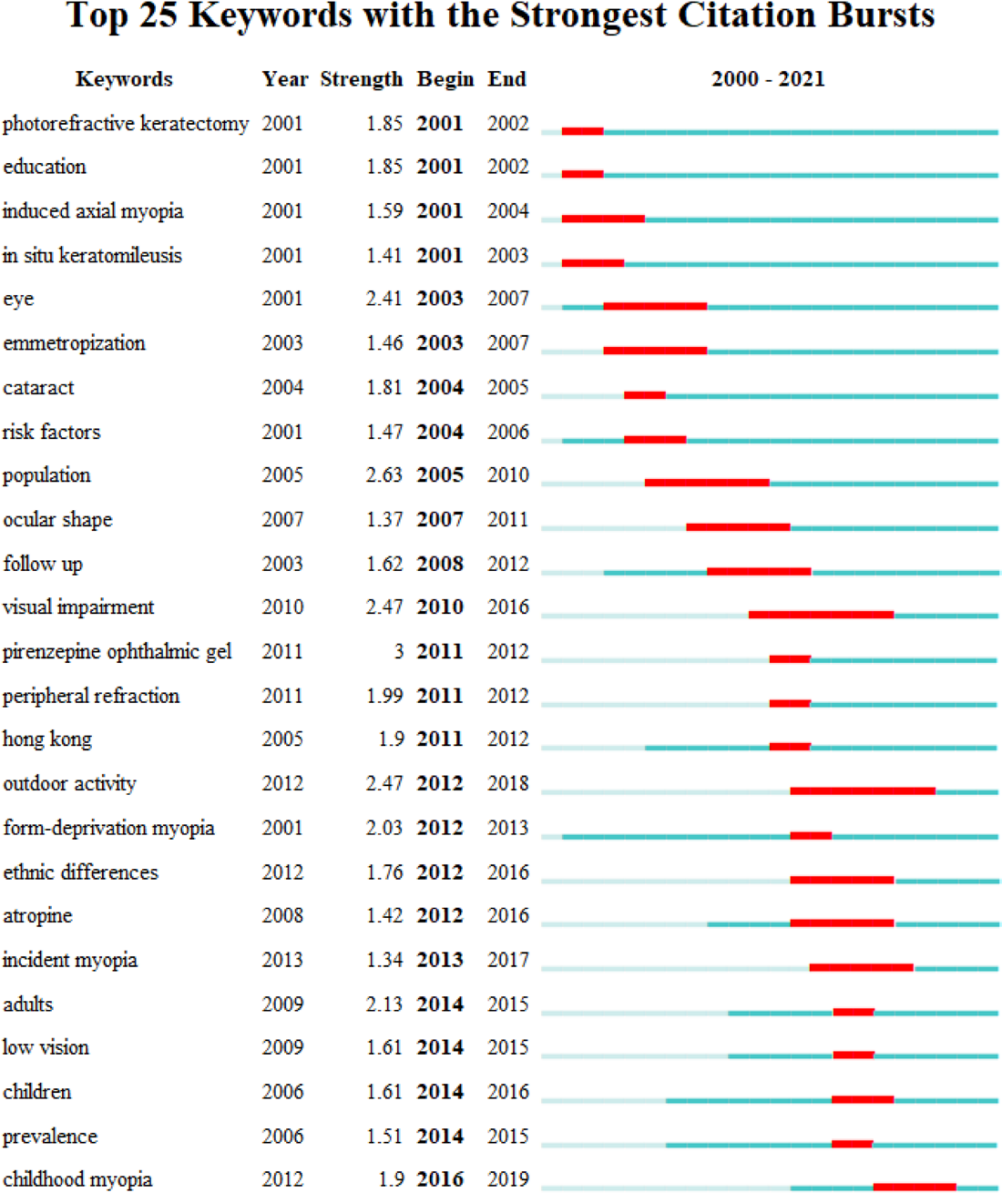
Top 25 keywords with the strongest citation bursts in the top 100 most cited papers.

Figures 8 and 9 dynamically depict the evolving trends of frontier hotspots in the field of myopia in the 21st century. Treatment modalities for myopia have consistently been a research hotspot, with a focus on pharmaceuticals, surgeries, and behavioral interventions. The outbreak of the corona virus disease 2019 pandemic and the rapid proliferation of online learning have significantly increased the frequency and duration of electronic device use among children and adolescents, leading to a sudden surge in myopia prevalence. Consequently, the pediatric population has become a focal point of myopia research in recent years. Additionally, emerging research topics on visual impairment and low vision reflect researchers’ profound concerns about the serious consequences stemming from the sustained progression of myopia. Cluster analysis results indicate that retinal detachment, form deprivation myopia, high myopia, in situ keratomileusis, and contrast sensitivity may be potential focal points and trends in future myopia research.

### 3.9. Co-cited articles and co-cited reference cluster analysis

**Figure 10.**
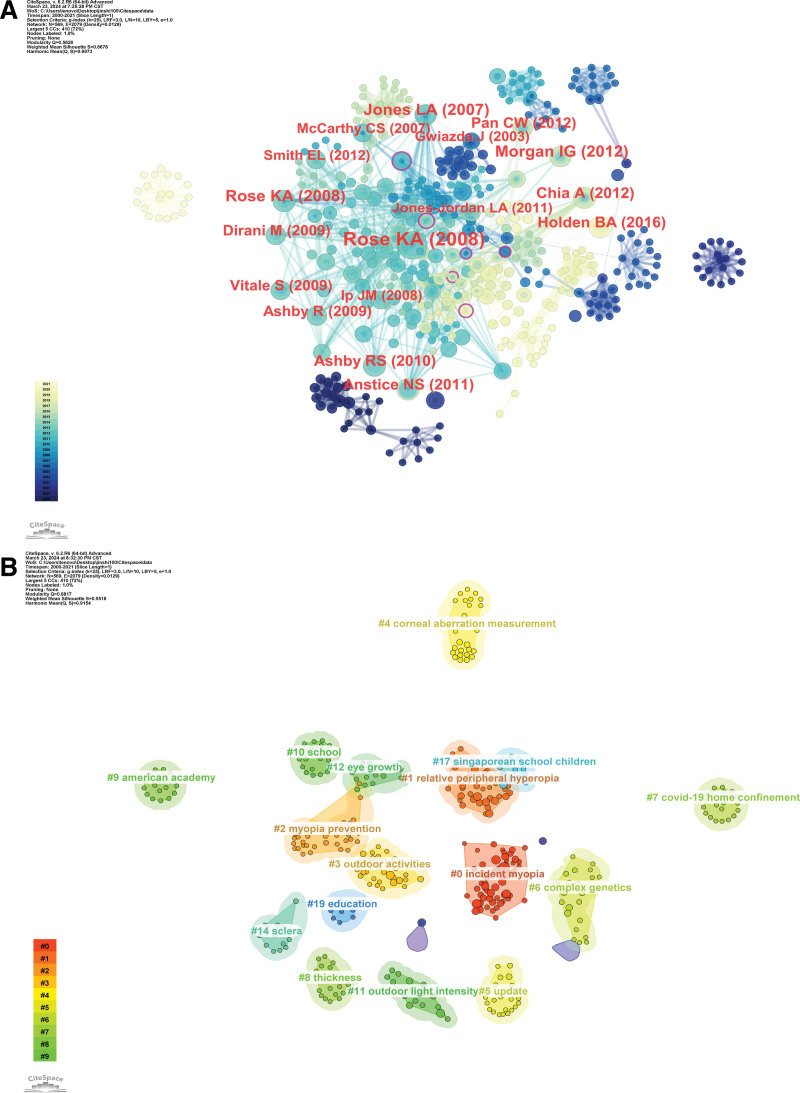
Network visualization and cluster analysis of co-cited references in the top 100 most cited papers.

**Figure 11.**
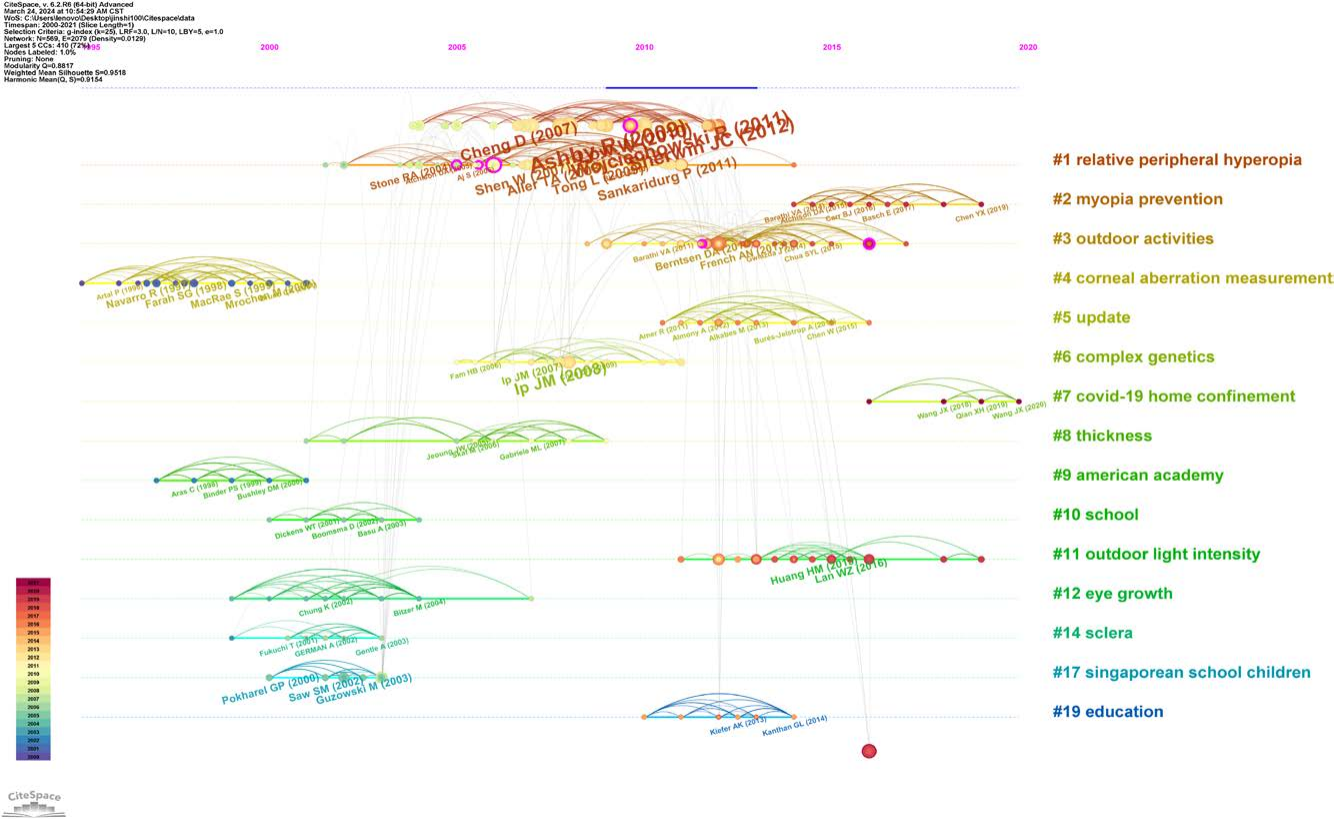
Visualization of clustering analysis of co-cited references in the top 100 most cited papers over time.

**Figure 12.**
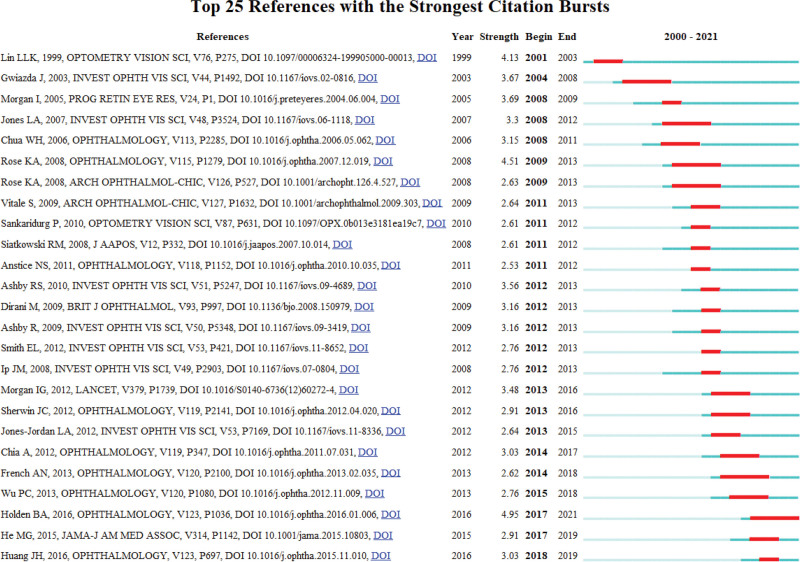
Top 25 references with the strongest citation bursts in the top 100 most cited papers.

When 2 or more papers are cited by one or more subsequent papers, a co-citation relationship is established, which evolves dynamically over time. Analyzing the co-citation network among references facilitates a comprehensive exploration of the developmental and evolutionary dynamics of a field. To achieve this, we utilized CiteSpace software to generate cluster networks and co-citation correlation analysis diagrams. Figure 10A shows the most frequently cited references. The 5 hot spots in myopia research are: #1 “incident myopia,” #2 “relative peripheral hyperopia,” #3 “myopia prevention,” #4 “outdoor activities,” and #5 “corneal aberration measurement” (Fig. 10B). Generally, Q > 0.3 indicates a significant clustering structure, while S > 0.5 suggests reasonable clustering results. A value of S > 0.7 is considered high-quality and convincing in academic research. In this study, the cluster diagram (Q = 0.8817, S = 0.9518) exhibited a highly significant clustering structure and demonstrated convincing quality. Figures 11 and 12 display the annual evolution of co-cited references.

Table 4 outlines the top 10 most cited references in the field of myopia. Among these, 6 studies specifically focus on the risk factors associated with myopia. Rose et al conducted 2 comprehensive studies revealing a significant positive correlation between increased outdoor activity time and reduced severity of myopia.^[30]^ They also highlighted educational pressure as a significant factor contributing to the increased prevalence of myopia among children.^[31]^ The research by Jones et al indicated that parental history of myopia was a crucial factor influencing the onset and progression of myopia in children.^[32]^ Specifically, there was a significant increase in the risk of myopia among children when both parents were myopic. Pan et al conducted a population-based study in children, revealing that the incidence of myopia varied by region and ethnicity.^[19]^ Additionally, they found that prolonged near-work activities, inadequate outdoor time, higher levels of education, and a family history of myopia were associated with an increased risk of myopia.^[19]^ Ashby et al observed changes in ocular biometric parameters in chicks exposed to different laboratory lighting conditions, finding that high levels of light per day effectively delayed the progression of experimental myopia.^[33]^ Subsequently, Ashby et al further demonstrated in a 2010 study that the protective effect of light against form-deprivation myopia was mediated by dopamine.^[34]^ In summary, the pivotal role of environmental factors in the development of myopia has been confirmed by the majority of studies. However, the etiology of myopia is complex and involves multifactorial interactions. Therefore, further in-depth exploration and research are necessary to comprehensively elucidate the pathogenesis of myopia.

**Table 4:** Top 10 most cited references in the myopia field.

Rank	Title	Journal	First author	Year	Citation
1	Outdoor activity reduces the prevalence of myopia in children	OPHTHALMOLOGY	Rose KA	2008	17
2	Myopia	LANCET	Morgan IG	2012	10
3	Parental history of myopia, sports and outdoor activities, and future myopia	INVEST OPHTH VIS SCI	Jones LA	2007	10
4	Myopia, lifestyle, and schooling in students of Chinese ethnicity in Singapore and Sydney	ARCH OPHTHALMOL—CHIC	Rose KA	2008	10
5	The effect of bright light on lens compensation in chicks	INVEST OPHTH VIS SCI	Ashby RS	2010	9
6	Atropine for the treatment of childhood myopia: safety and efficacy of 0.5%, 0.1%, and 0.01% doses (Atropine for the Treatment of Myopia 2)	OPHTHALMOLOGY	Chia A	2012	9
7	Effect of dual-focus soft contact lens wear on axial myopia progression in children	OPHTHALMOLOGY	Anstice NS	2011	9
8	Global Prevalence of Myopia and High Myopia and Temporal Trends from 2000 through 2050	OPHTHALMOLOGY	Holden BA	2016	9
9	The effect of ambient illuminance on the development of deprivation myopia in chicks	INVEST OPHTH VIS SCI	Ashby R	2009	8
10	Worldwide prevalence and risk factors for myopia	OPHTHAL PHYSL OPT	Pan CW	2012	8

## 4. Discussion

This study represents the first bibliometric analysis of the most influential literature in the field of myopia research in the 21st century. Due to the constant influx of papers into databases, researchers often struggle to efficiently identify key articles within their areas of interest. To address this challenge, we conducted a comprehensive analysis of the top 100 most cited articles related to myopia that were published since 2000, with the goal of helping researchers understand the patterns of clinical practice changes, current research frontiers, and future trends in this field.

### 4.1. General information from the top 100 most cited papers

In terms of article types, original articles make up over 80% of the top 100 cited papers, underscoring a pronounced inclination among researchers to reference original investigative endeavors. Notably, the most recently published article among these highly cited papers dates back to 2021. This study employed a prospective cross-sectional study design and compared the average spherical equivalent refraction and myopia prevalence in 2020 to those of the preceding 5 years. The findings shed light on the impact of home isolation during the corona virus disease 2019 pandemic on the progression of myopia in school-aged children.^[35]^

It is noteworthy that the combined citations of the first 20 papers account for nearly 40% of all citations, underscoring their significant impact. Through analysis of the authors in the literature, we identified a group of scholars dedicated to myopia research. Holden, BA, and Morgan, IG have emerged as the most influential authors in the field of myopia research in the 21st century, owing to their outstanding contributions. Additionally, we found that the article titled “Global Prevalence of Myopia and High Myopia and Temporal Trends from 2000 through 2050” by Holden, BA, garnered the highest total citation count and average yearly citations. This study shed light on the severity of the myopia issue on a global scale.^[4]^

Judging from the publication situation, the National University of Singapore stands out with exceptional productivity and citation impact, emerging as a leader in the field of myopia research. Additionally, institutions such as Chang Gung University, Kaohsiung Chang Gung Memorial Hospital, and Wenzhou Medical University have also made significant strides, establishing themselves as emerging forces in myopia research. *Investigative Ophthalmology & Visual Science* not only leads in the number of publications but also ranks first in total citations, affirming its authority and impact in the field of myopia research. Additionally, emerging myopia research journals such as *JAMA Ophthalmology*, *Nature Reviews Disease Primers*, and *Acta Ophthalmologic* have demonstrated robust growth momentum, providing researchers with further publication platforms.

From the perspective of geographical distribution, the United States, Australia, and China have emerged as primary centers for myopia research, collectively contributing to over two-thirds of the total literature count. Among them, the United States leads with 34 papers, highlighting its significant contributions and leadership, as well as providing insights into trends and focal points in global myopia research.

### 4.2. Future perspective in myopia research

Epidemiological investigations of myopia constitute the most researched topic among the top 100 most cited papers, addressing its distribution, progression, etiological mechanisms, and risk factors. Research has been conducted in various geographic regions, including Shanghai, Hong Kong, Taiwan, the United States, South Korea, Singapore, and Europe. Both genetic factors, such as parental myopia and racial differences, and environmental factors, including outdoor time, near work, education, and lighting conditions, influence the onset and progression of myopia. Although current research on the pathogenesis of myopia predominantly focuses on scleral hypoxia and dopamine mechanisms,^[11,36]^ the roles of choroidal thickness and neurotransmitters in myopia should not be overlooked. Studies indicated that the choroid is generally thinner in myopic patients and that choroidal thickness decreases with increasing myopia severity.^[37,38]^ This finding suggests that a reduction in choroidal thickness may serve as a biomarker for myopia progression and is associated with choroidal blood supply, nutrient delivery, and metabolic regulation. Therefore, monitoring and regulating choroidal thickness offers a new perspective for myopia intervention. Additionally, intraocular neurotransmitters, as crucial mediators of refractive development, influence retinal pigment epithelium growth and choroidal thickness, and play a significant role in scleral remodeling and myopia progression.^[39,40]^ Modulating neurotransmitter signaling pathways may represent a new strategy for myopia control. However, current epidemiological studies are limited by sample selection, insufficient environmental considerations, unclear genetic mechanisms, and inconsistent diagnostic criteria, all of which pose challenges to their accuracy and reliability. In the future, scholars should strengthen collaboration, further refine investigative methods, thoroughly consider the impact of various environmental factors, gain a deeper understanding of the pathogenesis of myopia, and advance the development of myopia prevention and control technologies.

Myopia treatment has received significant attention, as evidenced by 24 highly cited papers among the top 100 in the field. These studies detail various intervention measures for preventing and controlling myopia, as well as major advancements in refractive surgery throughout the 21st century. For example, Seiler et al (2000) found that photorefractive keratectomy corrects myopia and astigmatism but may increase postoperative aberrations affecting visual performance.^[41]^ Similarly, Marcos et al (2001) noted the correlation between laser-assisted in situ keratomileusis-induced aberrations and corneal surface changes.^[42]^ The 2004 Food and Drug Administration trial confirmed implantable collamer lenses surgery as safe and effective for moderate to high myopia.^[43]^ Sekundo et al introduced femtosecond lenticule extraction in 2008 and highlighted small incision lenticule extraction as a promising flapless procedure by 2011.^[44,45]^ Research indicates that progressive multifocal lenses, multifocal soft contact lenses, orthokeratology, and defocus incorporated soft contact lenses are more effective than single-vision glasses in slowing myopia progression. Additionally, randomized controlled trials show that 0.01% atropine eye drops are effective in slowing childhood myopia with fewer side effects than higher concentrations.^[22,46–49]^ These findings highlight the ongoing importance of clinical research in advancing myopia treatment. However, despite these advancements, further research and clinical trials are necessary to validate the effectiveness of current optical and pharmacological interventions.

In addition, research has explored incidence rates and treatment strategies for complications related to myopia, such as glaucoma, myopic choroidal neovascularization, myopic maculopathy, and retinal detachment.^[50–54]^ Optical coherence tomography plays a crucial role in predicting and evaluating myopia progression, as evidenced by its established role in the field.^[55,56]^ Recent studies have focused on myopia genomics, utilizing whole-genome association analysis to identify susceptibility genes and uncover potential genetic factors.^[57]^ Through genetic analyses, specific gene regions and pathways have been identified as being associated with myopia, highlighting the roles of extracellular matrix remodeling, the visual cycle, and neuronal development in its pathogenesis.^[58]^ These findings provide valuable insights into the pathogenesis of myopia and offer potential strategies for prevention and treatment.

## 5. Limitations

The study also has several limitations. Firstly, we primarily relied on the Web of Science Core Collection database, which may not encompass all relevant literature and could introduce minor result discrepancies due to regular updates. Our title keyword search method ensured precision, but it may have resulted in incomplete results. Restricting our search to English articles may have overlooked valuable studies in other languages, affecting the comprehensiveness of our findings. Manual exclusion of unrelated articles improved screening accuracy but might have missed some relevant literature. Lastly, while bibliometrics effectively reflects past research trends, it has limitations in identifying emerging hotspots and predicting future trends.

## 6. Conclusion

This study is the first bibliometric analysis of influential literature in 21st-century myopia research. It provides valuable insights into the evolution and current trends in the field by identifying leading papers, journals, and research directions, among others. The United States has contributed the most to this field, with Natl Univ Singapore publishing the largest number of papers. The majority of significant studies on myopia have been published in the journal *Investigative ophthalmology & visual science*. Since the beginning of the 21st century, significant advances have been made in the epidemiological characteristics and treatment of myopia, but the pathogenesis of myopia remains unclear. For example, the correlation between choroidal thickness and blood perfusion and myopia, as well as the effects of various neurotransmitters on myopia, still need to be further explored. This highlights the need for broad collaboration between different countries, institutions and academics, both domestically and internationally. By summarizing existing research hotspots and predicting future development trajectory, this study aims to help scholars to better understand the development of research on the etiology and pathogenesis of myopia and bridge gaps between existing knowledge and clinical practice. Ultimately, this can inform strategies to improve myopia management and treatment.

## 7. Recommendations

### *Establishing diagnostic and classification criteria*:

Current definitions and thresholds for various types of myopia continue to be controversial. Further research is necessary to establish harmonized diagnostic and classification criteria to enhance the communication and comparison of clinical trials and epidemiological results.

### Deepening the investigation into pathogenic mechanisms:

The current understanding of the physiological and pathological processes, as well as the signaling pathways involved in myopia, is limited. Research should focus on the pathogenic mechanisms of myopia, particularly scleral biology, changes in choroidal blood flow, and retinal morphology. This will enhance understanding of myopia and aid in developing targeted treatments.

### Exploring safe and effective treatment approaches:

Pharmacological interventions and refractive surgeries are effective but come with risks like dry eye syndrome and infection. It is recommended to explore safe and effective alternative therapies. These could be combined with conventional treatments to improve patients’ quality of life and myopia outcomes.

### *Building a personalized treatment system*:

Given the individual variability among myopic patients, some treatment modalities may show limited efficacy. Researchers are encouraged to tailor treatment approaches for each individual to achieve sustained improvements in visual health among myopic patients.

### Enhancing the evaluation system for treatment outcomes:

Currently, some myopia treatments lack standardized evaluation criteria and long-term follow-up studies. It is advisable to further refine the assessment system for treatment outcomes, enabling more objective and precise evaluations and comparisons of treatment efficacy.

### *Improving public awareness and education*:

The public currently lacks a thorough understanding of myopia’s etiology, risks, and preventive measures. Strengthening promotional efforts and educational campaigns to disseminate accurate eye care information and promote healthy lifestyle habits is advisable. These initiatives are crucial for improving public awareness and concern about myopia, particularly among adolescents, thus aiding in the preservation of visual health.

### Strengthening collaboration and interaction among multiple departments:

The current intensity and scope of myopia prevention and control work are still insufficient. It is recommended to enhance collaboration and interaction among families, communities, schools, and educational authorities, focusing on children’s eye habits. Regular vision screenings should be conducted to collectively foster a conducive environment for preserving visual health.

These recommendations aim to deepen understanding of myopia, strengthen preventive efforts, optimize diagnostic methods, and explore more effective treatment, with the hope of providing valuable reference and guidance for clinicians and researchers.

## Acknowledgments

The authors thank the software of Microsoft Office Excel 2019, CiteSpace 6.2.R6, VOSviewer 1.6.20, and Scimago Graphica1.0.41. We would like to appreciate the data availability provided through the WoSCC database. Finally, we would like to express our sincere gratitude to Chat-GPT 3.5 for providing language translation services for this study.

## Author contributions

**Data curation:** Qi Xun, Wenjing Mei.

**Formal analysis:** Qi Xun, Xuan Zhang.

**Funding acquisition:** Juan Yu.

**Supervision:** Yazheng Pang, Juan Yu.

**Visualization:** Qi Xun, Wenjing Mei.

**Writing – original draft:** Qi Xun, Wenjing Mei, Xuan Zhang.

**Writing – review & editing:** Yazheng Pang, Juan Yu.
